# Complete Genome Sequence of Lactobacillus acidophilus LA-10A, a Promising Probiotic Strain Isolated from Fermented Mare’s Milk

**DOI:** 10.1128/mra.00215-22

**Published:** 2022-05-09

**Authors:** Xueping Yu, Yang Yu, Jialiang Ouyang, Haixia Wen, Hongting Wang, Xin Ma

**Affiliations:** a Thankcome Biological Science and Technology (Suzhou) Co., Ltd., Suzhou, Jiangsu Province, China; b State Key Laboratory of Bioreactor Engineering, East China University of Science and Technology, Shanghai, China; University of Maryland School of Medicine

## Abstract

This report describes the whole-genome sequence of Lactobacillus acidophilus LA-10A, isolated from fermented mare’s milk. This strain has been widely consumed due to its excellent performance in the treatment and prevention of Helicobacter pylori infection. The genome sequence of LA-10A provides further molecular information about its features.

## ANNOUNCEMENT

Lactobacillus acidophilus is one of the commercially significant probiotics, originally isolated from the human gastrointestinal tract and designated Bacillus acidophilus in 1900 (1). L. acidophilus has been reported to play a pivotal role in maintaining human health by alleviating lactose intolerance (2), nonalcoholic fatty liver disease (3), irritable bowel syndrome (4), and hypercholesterolemia (5) and preventing Helicobacter pylori infection (6, 7). Strain LA-10A was first isolated in 2016 from homemade fermented mare’s milk that was prepared by a shepherd in Xinjiang, China. A sample (1 g) was serially diluted and spread onto MRS agar, then incubated under anaerobic conditions at 37°C for 48 h. One white colony growing on the plates was isolated and identified as Lactobacillus acidophilus by 16S rRNA gene sequencing. L. acidophilus LA-10A was deposited at the China Center for Type Culture Collection (CCTCC number M2019012).

A single colony of strain LA-10A was inoculated into MRS medium and cultured at 37°C for 48 h. Subsequently, high-quality genomic DNA was extracted using a modified cetyltrimethylammonium bromide (CTAB) method (8, 9). Genomic DNA was sheared using g-TUBEs, then end-repaired to prepare SMRTbell DNA template libraries according to the manufacturer’s instructions. The library quality was analyzed using Qubit, and the average fragment size was estimated using an Agilent 2100 Bioanalyzer. Single-molecule real-time (SMRT) sequencing was performed using the PacBio Sequel II platform. The polymerase reads generated were processed using SMRTLink v8.0, with the parameter minLength set to 50 and all other parameters kept at default. One SMRT cell produced a total of 4.47 Gb, including 533,696 subreads (average length, 8,381 bp) with an *N*_50_ value of 8,657 bp; *de novo* assembly was conducted using Canu v2.0 (10, 11). Default parameters were used for all software unless otherwise specified.

The genome was assembled using Canu v2.0 with the parameters described in our previous paper (12), and the results showed it to be a circularized structure with no plasmids. Canu detects and annotates the final assembly as circular when the best overlap graph (BOG) is circular. The average coverage depth was over 93-fold. The repeated sequences at the two ends identified by BLASTN v2.7.1 confirmed the chromosome circularization. The assembly sequence was reordered such that the beginning of the assembly started with the *dnaA* gene, yielding a single contig of 1,991,561 bp corresponding to a complete and circular chromosome (mean G+C content, 34.71%). Genome annotation was carried out subsequently using the NCBI Prokaryotic Genome Automatic Annotation Pipeline (PGAP) (13). A total of 1,896 protein-coding genes (CDSs), 12 rRNAs, and 61 tRNAs were predicted. Of the CDSs, 82.65% were classified into at least one Cluster of Orthologous Groups (COG), and 54.85% were classified into at least one Kyoto Encyclopedia of Genes and Genomes (KEGG) orthology region. Genes known to confer resistance to antibiotics of human or veterinary importance were not found in the genomic sequences, nor were toxin genes or hazardous virulence factors, indicating the safety of strain LA-10A.

### Data availability.

The complete genome sequence of Lactobacillus acidophilus LA-10A is available at NCBI GenBank under Sequence Read Archive (SRA) accession number SRR18189666. The raw sequence reads have been deposited in the SRA under BioProject accession number PRJNA810352 and BioSample accession number SAMN26264341.
